# Video‐Assisted Thoracoscopic Right Upper Lobe Sleeve Resection After Endobronchial Treatment of Bronchial Carcinoid: Case Report

**DOI:** 10.1155/carm/2079223

**Published:** 2026-01-29

**Authors:** Yangbo Qiu, Junjun Ni, Rui Li, Minglei Yang

**Affiliations:** ^1^ Department of Thoracic Surgery, Ningbo No. 2 Hospital, Ningbo, China, nbws.gov.cn

**Keywords:** bronchial carcinoid, endobronchial treatment, right upper lobe sleeve resection, video-assisted thoracoscopic surgery

## Abstract

A 47‐year‐old woman presented with significant respiratory symptoms and was diagnosed with a bronchial tumor. Histological analysis confirmed the tumor, initially treated with endobronchial treatment (EBT), was a typical carcinoid. Given the patient’s improved pulmonary function and in accordance with current clinical guidelines, she underwent video‐assisted thoracoscopic right upper lobe sleeve resection for the residual tumor. The combination of surgical resection and EBT may offer an effective treatment strategy for other bronchial tumors.

## 1. Introduction

The 2021 World Health Organization (WHO) classification of lung tumors groups neuroendocrine neoplasms (NENs) into typical carcinoid (TC), atypical carcinoid (AC), large cell neuroendocrine carcinoma (LCNEC), and small cell carcinoma (SCLC) [1]. TCs are less aggressive, with 5‐ and 10‐year survival rates of 87%–100% and 82%–87%, respectively, whereas ACs show more malignant behavior, with survival rates of 50%–95% and 38%–75% [2]. Surgical resection, including lobectomy or parenchyma‐sparing techniques (e.g., segmentectomy and sleeve lobectomy), is standard for localized TC or AC [3]. Endobronchial treatments (EBTs) may be considered in selected cases [4]. We present a case of a 47‐year‐old woman with a right upper lobar bronchus carcinoid, treated with video‐assisted thoracoscopic (VATS) right upper lobe sleeve resection after bronchoscopic partial resection.

## 2. Case Presentation

A 47‐year‐old never‐smoking female presented with a 3‐month history of persistent cough and 1‐month history of mild hemoptysis (< 5 mL). Physical examination revealed wheezing. Routine blood tests were unremarkable. A chest computed tomography (CT) and bronchoscopy confirmed a 16‐mm round lesion almost completely obstructing the right main bronchus. Pulmonary function tests revealed forced expiratory volume in one second (FEV1) of 0.93 L (actual), 2.07 L (predicted), and 45% of predicted. Severe obstruction and mild reduction in the carbon monoxide diffusion capacity (DLCO) were noted, making surgical resection inappropriate. To obtain a pathological diagnosis, alleviate symptoms, and improve lung function, the patient underwent combined rigid and flexible bronchoscopy. A well‐vascularized polypoid lesion was resected (tumor tissue fragmented into 7 × 4 × 3 mm to 12 × 12 × 5 mm pieces), with residual tumor obstructing the right upper bronchus. Histopathology confirmed a neuroendocrine tumor, consistent with a TC, with mitotic figures < 2 per HPF and Ki‐67 < 5%.

**Figure 1:**
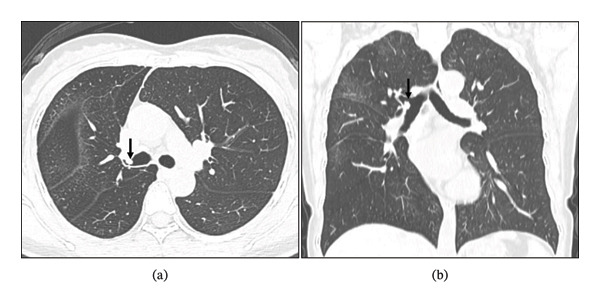
(a) CT scan showing a 6‐mm tumor (black arrow) at the anterior wall of the right upper bronchus (maximum diameter in the horizontal plane). (b) The tumor (black arrow) is located more than 20 mm from the right middle bronchus (or the right lower bronchus).

**Figure 2:**
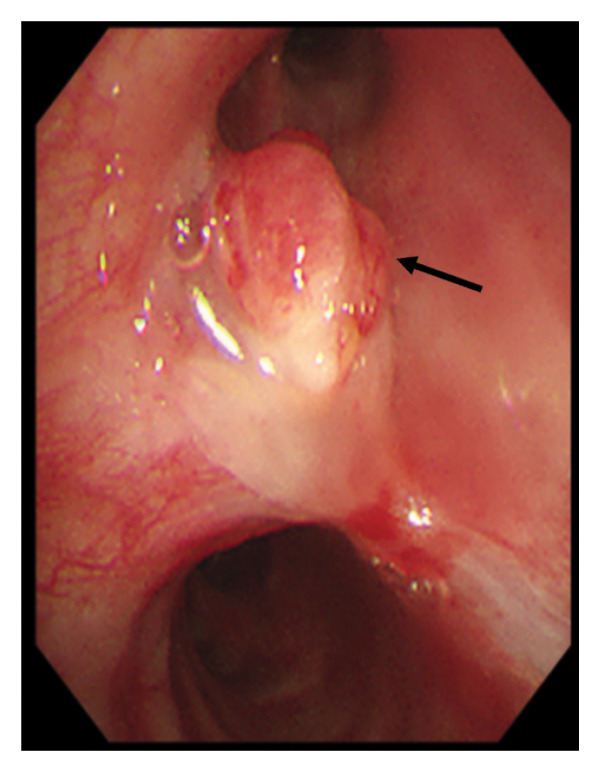
Bronchoscopy showing a well‐vascularized residual tumor (black arrow) at the orifice of the right upper bronchus.

**Figure 3:**
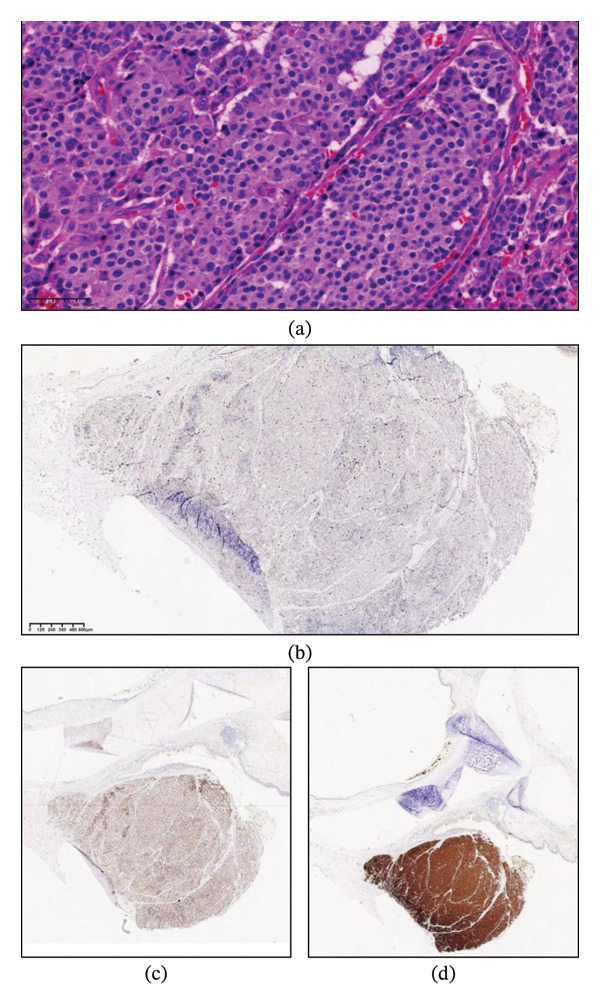
(a) Histopathology showing typical carcinoid: The tumor is composed of uniform cells with round or polygonal nuclei and inconspicuous nucleoli. No mitotic figures or areas of necrosis were observed (hematoxylin and eosin stain, × 40). (b) Ki‐67 immunostaining showing tumor cells with < 5% positivity. (c) INSM1 expression in tumor cells. (d) Synaptophysin expression in tumor cells.

**Figure 4:**
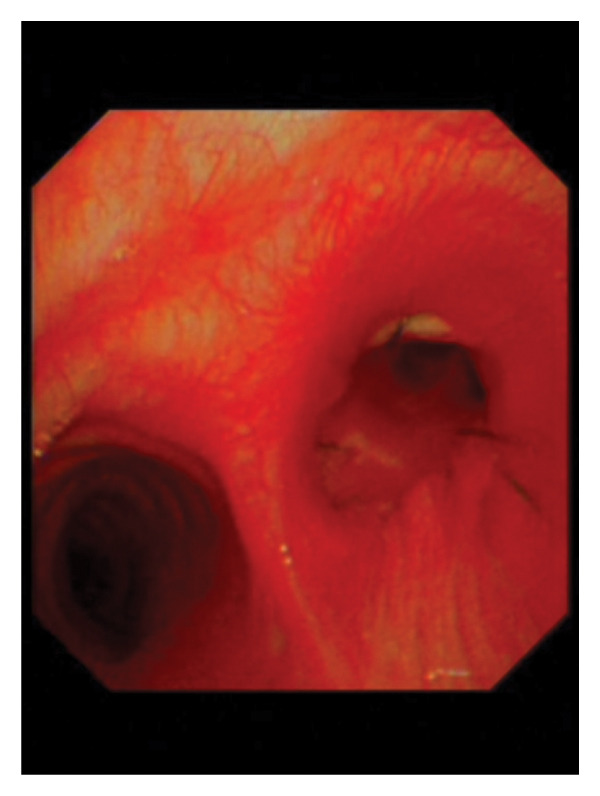
Follow‐up bronchoscopy performed 1 month after surgery showing no evidence of local recurrence of the bronchial carcinoid.

Following consultation with the thoracic surgery department and review of guidelines [3, 5], the patient was advised chemotherapy but not offered surgical resection for the residual tumor. As the local hospital did not approve further surgical intervention, the patient presented to our hospital 4 weeks later for resection of the residual tumor. Upon readmission, physical examination revealed mild wheezing, and blood tests were normal. Cardiopulmonary exercise testing showed an FEV1 of 2.08 L (97% predicted) and a peak oxygen consumption (VO2peak) of 18.3 mL/kg/min, indicating moderate perioperative risk but suitability for surgery. CT showed a 6‐mm tumor at the anterior wall of the right upper bronchus (Figure 1(a)), with no involvement of the right middle or lower bronchi (Figure 1(b)). Bronchoscopy confirmed a well‐vascularized residual tumor at the orifice of the right upper bronchus (Figure 2). After preoperative evaluation, the patient underwent VATS right upper lobe sleeve resection with lymphadenectomy through a 4‐cm incision. Intraoperative histopathology showed clear margins. Final pathology confirmed a 7‐mm TC with no lymph node metastasis. Immunohistochemistry was positive for INSM1, Syn, and Ki‐67 (< 5%) (Figure 3). The patient was discharged on postoperative day 9. Follow‐up bronchoscopy at 1 month showed no evidence of recurrence (Figure 4). CT scans at 6 months and 1 year further demonstrated no tumor recurrence, and the patient remains asymptomatic to date.

## 3. Discussion

In our patient, we opted for VATS right upper lobe sleeve resection instead of continued observation, chemotherapy, or further EBT for the residual tumor for three reasons. First, preoperative chest CT and bronchoscopy confirmed the residual tumor at the orifice of the right upper bronchus, which was highly vascular. Thus, the possibility of tumor progression required intervention. Second, prior EBT confirmed the tumor as a TC, and current clinical guidelines support surgery as the primary treatment for localized, resectable TCs and ACs, with adjuvant therapy still debated. The European Neuroendocrine Tumor Society (ENETS) recommends surgery as the only curative option and restricts adjuvant therapy to patients with AC and positive lymph nodes [3, 5]. After previous EBT, preoperative assessments indicated suitability for surgical resection with manageable perioperative risk. Third, considering the patient’s age, well‐preserved pulmonary function (preoperative FEV1: 2.08 L, 97% of predicted), and tumor location, VATS right upper lobe sleeve resection with lymphadenectomy was the ideal approach.

EBT, including laser therapy, cryotherapy, and argon plasma coagulation, is an evolving technology for both diagnostic sampling and complete resection of pulmonary carcinoids, with successful removal of microscopic residuals. Recent studies defined successful EBT as the absence of residual disease for 2 years posttreatment, confirmed by CT and bronchoscopy. These studies identified patients with intraluminal carcinoid tumors < 20 mm in diameter as good candidates for EBT, while those with tumors ≥ 20 mm should undergo surgery [6]. Although our patient met the criteria for EBT, residual tumor persisted at the right upper bronchial orifice after a single session, and the patient declined further EBT. Thus, EBT efficacy could not be evaluated per the aforementioned criteria. However, EBT was crucial for bronchial deobstruction and reducing the extent of subsequent resection.

In conclusion, combining surgical resection with EBT offers an alternative strategy for treating bronchial carcinoids. In addition to providing radical disease control, it offers locoregional benefits, such as deobstruction of the involved bronchus and improvement in pulmonary function, making it an ideal therapeutic approach. The combined approach of surgery, EBT, and other emerging technologies (e.g., photodynamic therapy) may also be applicable for the management of bronchial tumors, including bronchial carcinoids.

## Funding

This study was supported by the Ningbo Top Medical and Health Research Program, 2022030208.

## Ethics Statement

This case report complies with ethical guidelines and adheres to local legal requirements.

## Consent

No identifying information other than age and gender is included in this manuscript. Written informed consent for the use of all clinical data and imaging materials related to this case has been obtained from the patient and their family. All authors of this article have consented to its publication.

## Conflicts of Interest

The authors declare no conflicts of interest.

## Data Availability

The datasets used and analyzed in this study are available from the corresponding author upon reasonable request.
